# Proteogenomic analysis dissects early-onset breast cancer patients with prognostic relevance

**DOI:** 10.1038/s12276-024-01332-w

**Published:** 2024-11-01

**Authors:** Kyong-Ah Yoon, Youngwook Kim, So-Youn Jung, Jin-Sun Ryu, Kyung-Hee Kim, Eun-Gyeong Lee, Heejung Chae, Youngmee Kwon, Jaegil Kim, Jong Bae Park, Sun-Young Kong

**Affiliations:** 1 Department of Biochemistry, College of Veterinary Medicine, Konkuk University, Seoul, Korea; 2 Department of Cancer Biomedical Science, Graduate School of Cancer Science and Policy, National Cancer Center, Goyang, Korea; 3 Center for Breast Cancer, National Cancer Center, Goyang, Korea; 4 Division of Translational Science, Research Institute, National Cancer Center, Goyang, Korea; 5 Laboratory Animal Research Facility, Research Institute, National Cancer Center, Goyang, Korea; 6 Proteomics Core Facility, Research Core Center, Research Institute, National Cancer Center, Goyang, Korea; 7 Cancer Data Center, Control Institute, National Cancer Center, Goyang, Korea; 8 Division of Medical Oncology, Hospital, National Cancer Center, Goyang, Korea; 9 GlaxoSmithKline, Waltham, MA USA; 10 Department of Laboratory Medicine, Research Institute, National Cancer Center Korea, Goyang, Korea; 11 Department of Targeted Therapy Branch, Research Institute, National Cancer Center, Goyang, Korea

**Keywords:** Cell signalling, Breast cancer

## Abstract

Early-onset breast cancer is known for its aggressive clinical characteristics and high prevalence in East Asian countries, but a comprehensive understanding of its molecular features is still lacking. In this study, we conducted a proteogenomic analysis of 126 treatment-naïve primary tumor tissues obtained from Korean patients with young breast cancer (YBC) aged ≤40 years. By integrating genomic, transcriptomic, and proteomic data, we identified five distinct functional subgroups that accurately represented the clinical characteristics and biological behaviors of patients with YBC. Our integrated approach could be used to determine the proteogenomic status of HER2, enhancing its clinical significance and prognostic value. Furthermore, we present a proteome-based homologous recombination deficiency (HRD) analysis that has the potential to overcome the limitations of conventional genomic HRD tests, facilitating the identification of new patient groups requiring targeted HR deficiency treatments. Additionally, we demonstrated that protein–RNA correlations can be used to predict the late recurrence of hormone receptor-positive breast cancer. Within each molecular subtype of breast cancer, we identified functionally significant protein groups whose differential abundance was closely correlated with the clinical progression of breast cancer. Furthermore, we derived a recurrence predictive index capable of predicting late recurrence, specifically in luminal subtypes, which plays a crucial role in guiding decisions on treatment durations for YBC patients. These findings improve the stratification and clinical implications for patients with YBC by contributing to the optimal adjuvant treatment and duration for favorable clinical outcomes.

## Proteogenomic analysis reveals subgroups in young breast cancer patients

Breast cancer is the most common cancer in women globally, with a significant number of young breast cancer (YBC) cases in Korea. YBC is known for its aggressive nature and poor outcomes. This study is aimed to understand the molecular features of YBC in Korean patients using a multiomics approach, which involves analyzing genomic, transcriptomic, and proteomic data from 178 young women with breast cancer. They examined tumor samples and patients’ blood to identify molecular signatures and pathways that could predict clinical outcomes. The team found unique molecular profiles in YBC, including higher mutation rates in certain genes and unique gene expression patterns. They identified several key pathways and potential biomarkers that could predict the cancer’s aggressiveness and the patients’ prognosis. The study revealed differences in the tumor environment and immune response, which could affect treatment strategies.

This summary was initially drafted using artificial intelligence, then revised and fact-checked by the author.

## Introduction

Breast cancer is the most common malignancy among women globally^1^ and is the leading cancer among women in Korea^2^. The occurrence of young breast cancer (YBC), i.e., breast cancer in patients aged 40 years or younger at diagnosis, is rare; however, its proportional incidence in Korea is approximately 10%^3^, which is twice the incidence rate in the US^4^. Patients with YBC have a genetic susceptibility caused by *BRCA1/2* pathogenic variants, showing aggressive clinical phenotypes with advanced stages, high-grade and triple-negative breast cancer (TNBC), and an unfavorable prognosis, including late recurrence^5–8^. However, the molecular features of YBC underlying these more aggressive tumor characteristics and worse clinical outcomes are unclear. Several studies have revealed the characteristics of YBC. With respect to somatic mutations, the Cancer Genome Atlas (TCGA) and Molecular Taxonomy of Breast Cancer International Consortium (METABRIC) databases revealed that premenopausal (PreM) patients with breast cancer have greater numbers of mutations in 5 genes—*CDH1*, *GATA3*, *MLL3*, *GPS2*, and *PI3KCA*. Moreover, compared to post-menopausal (PostM) tumors, gene expression in PreM tumors is enriched in the integrin and laminin signaling pathways, EGFR signaling activation, and TGF-β, especially in estrogen receptor-positive (ER + ) breast cancer^9^. Another study revealed that, compared with Western patients, Asian PreM patients presented a characteristic increase in tumor-infiltrating lymphocytes (TILs) and a decrease in TGF-β signaling, which suggested that Asian YBCs harbor a more immune-active microenvironment^10^. An age-specific gene expression study of breast cancer in Middle Eastern women identified 63 genes that were specific to YBC ( ≤ 45 years) and 2 genes (*TIAM1* and *VANGL2*) whose expression was significantly lower in breast tumors of very young ( ≤ 35 years) women than in breast tumors from other age groups^11^.

In recent studies, the application of proteogenomic (PG) analysis has improved our understanding of breast cancer by providing comprehensive molecular signatures relevant to clinical features^12–14^. Stromal-enriched clusters, G protein-coupled receptor clusters, and several targetable biological pathways were also identified. Although this approach revealed new clusters and refined subgroups with potential clinical benefits in breast cancer, there is a need to investigate the molecular landscape in young Asian patients.

Therefore, this study aimed to evaluate and analyze the molecular characteristics of YBC in Korean patients (≤40 years) via integrated multiomics studies, including genomic, transcriptomic, and proteomic features, and to identify pathways for predicting the clinical outcomes of patients with YBC.

## Materials and Methods

### Study objectives and specimens

A total of 178 participants with histologically defined breast cancer, aged 40 years or younger, treated at the National Cancer Center (NCC) in Korea were included in this study. Tumor and adjacent normal tissue samples were obtained from surgically resected specimens, and blood samples were collected from the patients. This study was approved by the Institutional Review Board of the NCC (IRB nos. NCCNCS 13717, NCC2017-0127, and NCC2020-0135). Since some patients had limited samples, only 126 patients for whom all proteogenomic data were fully generated were used for analysis. We retrospectively reviewed the medical and pathological records of the patients, including histological diagnoses of surgical specimens, tumor staging, histological grade, treatment history (type of surgery, use of chemotherapy, hormone therapy, anti-HER2 therapy, and radiotherapy), and follow-up data (recurrence, metastasis, and death).

Tissue specimens and blood samples were provided by the NCC Bio Bank of the National Cancer Center, Korea. The frozen tissue samples were weighed and washed with cold phosphate-buffered saline (PBS) to remove blood contamination, placed in a tube (Covaris, Woburn, MA, USA), snap-frozen in liquid nitrogen, and pulverized using a cryoPREP tissue disruption system (CP02; Covaris). The pulverized tissue powder was divided into 10–20 mg aliquots for DNA, RNA, and protein extraction. Genomic DNA and RNA were extracted from cryopreserved tissue samples and blood samples using the AllPrep DNA/RNA Mini Kit, the QIAamp DNA Blood Mini Kit, the DNeasy Blood & Tissue Kit, and the RNeasy Micro Kit (Qiagen, Valencia, CA, USA) according to the manufacturers’ instructions. The concentration and integrity of the extracted RNA were assessed via a NanoDrop spectrophotometer (Thermo Fisher Scientific, Waltham, MA, USA) and an Agilent 2100 Bioanalyzer (Agilent Technologies, Santa Clara, CA, USA).

### Whole exome sequencing (WES) analysis

To generate standard exome capture libraries, the SureSelect Human All Exon V6 + UTR probe set (Agilent Technologies) was used according to the manufacturer’s instructions. Briefly, genomic DNA extracted from the samples was fragmented and processed for adaptor ligation and PCR amplification. The exome capture libraries were constructed and amplified. The final purified products were quantified according to the qPCR Quantification Protocol Guide (KAPA Library Quantification kits for Illumina Sequencing platforms) and qualified using a TapeStation DNA Screentape D1000 (Agilent). The products were sequenced using the NovaSeq platform (Illumina Inc., San Diego, CA, USA). Quality-calibrated fastq files were aligned to the reference genome hg19 using BWA-MEM^15^. Each read group was aligned to the reference genome separately, and all read group alignments that belonged to a single aliquot were merged using Picard Tools, SortSam and MergeSamFiles. Duplicate reads were flagged to prevent downstream variant call errors. Local realignment of insertions and deletions was performed using GATK:IndelRealigner^16^. A base quality score recalibration (BQSR) step was then performed using GATK:BaseRecalibrator^17^. Somatic mutation detection was performed via MuTect and GATK:Mutect2^18^, and the mutations were annotated with an oncotator^19^. The GATK Haplotyper caller was used to detect germline variants^20,21^.

### Whole-transcriptome sequencing (WTS)

Only high-quality RNA (RNA integrity ≥ 7.0) generated using the Illumina TruSeq Stranded mRNA Sample Prep Kit (Illumina) was used for RNA library construction. Qualified libraries were indexed, and paired-end 100-bp reads were sequenced using the Illumina NovaSeq platform. The quality of the sequencing reads was evaluated and examined for primer/adaptor sequence contamination via FastQC (v0.11.7)^22^. Trim Galore^23^ was employed for trimming the reads, applying a threshold of an average sequence quality > 30. The trimmed reads were subsequently aligned to the human reference genome using STAR (STAR-2.7.0). Gene expression profiling was conducted with the HTseq-count algorithm, which uses the raw read counts as input data. Finally, edgeR was used to convert the count data to reads per kilobase million (RPKM), or the transcript per million (TPM) algorithm from the bioinfokit Python package (v1.0.4)^24^ was used to adjust different sequencing throughputs between the samples.

### Proteomic analysis

Proteomic analysis was performed according to the standard protocol reported by the Clinical Proteomic Tumor Analysis Consortium (CPTAC)^25^. Briefly, cryopreserved tissue powder samples were solubilized in sodium dodecyl sulfate (SDS) solubilization buffer (5% SDS, 50 mM TEAB; pH 8.5) using an S220 focused ultrasonicator (Covaris). Proteins were digested using S-Trap™ spin columns (ProtiFi, Huntington, NY, USA), cleaned with C18 spin columns (Thermo Fisher Scientific, Rockford, IL, USA), and the desalted peptide samples were labeled using TMT11plex reagents (Thermo Fisher Scientific). Pooled 11-plex tandem mass tag (TMT)-labeled samples were processed for peptide fractionation using an Agilent 1260 Infinity HPLC system (Agilent)^26^ and then prepared for global proteome and phosphoproteome analyses. Phosphopeptide enrichment was performed using immobilized metal affinity chromatography^27^. The TMT-labeled peptides were loaded onto a trap column (Acclaum PepMap^TM^ 100, 75 mm × 2 cm), separated on an analytical column (EASY-Spray column, 75 mm × 50 cm; Thermo Fisher Scientific) via an Ultimate 3000 RSLCnano system (Thermo Fisher Scientific), and analyzed using the top 10 data-dependent methods using a Q Exactive HF-X hybrid quadrupole–orbitrap mass spectrometer (Thermo Fisher Scientific).

### Cell lines and reagents

Human breast cancer cell lines were cultured according to the distributors’ instructions. We obtained breast cancer cell lines from the American Type Culture Collection (ATCC) in Manassas, Virginia, USA; the Korean Cell Line Bank in Seoul, Korea; and the DSMZ in Braunschweig, Germany. MDA-MD-453, BT-474, and HCC-1954 cells were maintained in RPMI 1640 medium supplemented with 10% fetal bovine serum (FBS). SKBR3 cells were cultured in McCoy’s 5A medium supplemented with 10% FBS. JIMT-1 cells were maintained in Dulbecco’s modified Eagle’s medium (DMEM) supplemented with 10% FBS. Penicillin/streptomycin (1%) was added to all of the culture media, and the cells were cultured at 37 °C with 5% CO2.

### Droplet digital PCR (ddPCR)

The copy number of the *HER2* gene was analyzed using droplet digital PCR (ddPCR) on a QX200 Droplet Digital PCR System by Bio-Rad Laboratories. Fluorescent probes (FAM and HEX) were prepared from the PrimePCR^TM^ ddPCR^TM^ Copy Number Assay for ddPCR (dHsaCP1000116 for *HER2*, dHsaCP2500349 for *EIF2C1*, and dHsaCNS516206038 for *POLR2A* as the reference control) (Bio-Rad Laboratories Inc., Hercules, CA, USA). A total of 20 µl of PCR mixture was prepared with 10 ng of genomic DNA using 2X ddPCR supermix for the probe (10 µl) and 20X *HER2* and *EIF2C1* or *POLR2A* probes (FAM/HEX) (1 µl). PCRs were run on a Mastercycler nexus gradient Thermal Cycler (Eppendorf, Hamburg, Germany) at 95 °C for 10 min, followed by 40 cycles of 94 °C for 30 s, 55 °C for 60 s, and a 10 min incubation at 98 °C. Then, the PCR plates were read with a Bio-Rad QX200 droplet reader (Bio-Rad Laboratories, Inc.) and QuantaSoft^TM^ version 1.4.0 software (Bio-Rad Laboratories, Inc.) to assess the number of droplets positive for *HER2* and *EIF2C1* or *POLR2A*.

### Viability assay

Breast cancer cell lines were seeded in triplicate at 2–4 × 10^3^ cells per well, depending on the cell line, in 96-well plates. We also treated breast cancer cell lines with neratinib (Selleckchem, Houston, TX, USA) or lapatinib (Selleckchem). After 48 h of treatment with the test compounds, cell viability was examined using a Cell Counting Kit-8 (CCK-8, Dojindo Laboratories, Kumamoto, Japan). Dimethyl sulfoxide (DMSO) (0.1%) was used as the control. The half-maximal inhibitory concentration (IC_50_) was analyzed using GraphPad Prism (version 5.03, GraphPad Software, San Diego, CA, USA).

### Western blot

Total protein was extracted from cell lines or tissues using RIPA lysis buffer (Thermo Fisher Scientific) with protease and phosphatase inhibitor cocktails (GenDEPOT, Baker, TX, USA) following the manufacturer’s instructions. The protein concentrations were determined via a protein assay solution from Bio-Rad Laboratories. Proteins were separated via sodium dodecyl sulfate‒polyacrylamide gel electrophoresis (SDS‐PAGE), and the separated proteins were transferred to polyvinylidene fluoride (PVDF) membranes (Merck Millipore, Billerica, MA, USA). The blots were then blocked for 1 h with 5% skim milk (BD Biosciences, San Diego, CA, USA) in Tris-buffered saline containing 0.1% Tween 20 and incubated overnight with the indicated primary antibodies. Antibodies against the following proteins were used: phospho-HER2-Tyr1248 (Cell Signaling Technology, CST), HER2 (Abcam, Cambridge, UK), phospho-HER2-Ser1054 (Thermo Fisher Scientific), and β-actin (CST, used as the loading control). Proteins were visualized via a horseradish peroxidase-conjugated secondary antibody (CST) and an enhanced chemiluminescence (ECL) reagent (Bio-Rad Laboratories).

### Multiplex immunofluorescence staining

Multiplex immunofluorescence staining for cytokeratin (CK), PD-L1, CD68, CD8, FOXP-3, and PD-1 proteins was performed using a Leica Bond Rx Automated Stainer (Leica Biosystems, Newcastle, UK). Slides with tumor tissue sections were incubated with antibodies against the following proteins: CK (NBP2-29429; Novus Bio; dilution 1:300), PD-L1 (136845; CST; dilution 1:300), CD68 (ab192847; Abcam; dilution 1:300), CD8 (MCA1817; Bio-Rad; dilution 1:300), FOXP3 (ab20034; Abcam; dilution 1:100), and PD-1 (ab137132; Abcam; dilution 1:500). Then, the sections were incubated with the Polymer HRP Ms+Rb (ARH1001EA, AKOYA Biosciences) and subsequently treated with an Opal fluorophore (Opal690, Opal570, Opal520, Opal620, Opal480, or Opal780). Nuclei were subsequently visualized with DAPI, and the sections were coverslipped with ProLong Gold antifade reagent (P36934, Invitrogen). The slides were scanned using the Vectra Polaris Automated Quantitative Pathology Imaging System (Akoya Biosciences, Marlborough, MA, USA), and regions of interest were selected for all available tumor-containing areas. Images were analyzed using Inform 2.5 software (Akoya Biosciences, Marlborough, MA, USA) and TIBCO Spotfire™ (TIBCO, Palo Alto, CA, USA).

### Homologous recombination deficiency (HRD) score

The HRD score was determined as a simple sum of three factors: NtAI (number of telomeric allelic imbalances), LST (large-scale state transitions), and HRD-LOH (HRD loss of heterozygosity). Tumors with HRD scores ≥ 42 were defined as HRD-high. For the determination of HRD scores via WES, BAM files of tumor samples were applied to the Sequenza followed by the scarHRD and/or HRDetect package using the default parameters as previously described^28–30^.

### Differentially Expressed Gene (DEG) analyses

DEG analyses were conducted using edgeR^31^, Cuffdiff2^32^, and DESeq2^33^ following previously published protocols with default parameters. edgeR employed generalized linear models with tagwise dispersion, and raw counts were used as the primary input. As Cuffdiff2 does not work with count matrices, aligned transcriptomic reads were assembled into transcripts using Cufflinks with quartile normalization, bias correction, multiread correction, and a reference gene model. Cuffdiff2 identified DEGs via geometric library normalization and per-condition dispersion estimation. Adjusted p values were calculated with Benjamini‒Hochberg false discovery correction (5%) for all methods.

### Significance of mutation

The MutSigCV algorithm (v.1.3.5)^34^ was employed to discern significantly mutated genes in our cohort. The default parameters of three inputs, coverage table files, covariate table files and mutation type dictionaries, were used to run MutSigCV, which uses a statistical framework to evaluate the observed mutation patterns against background mutation rates while considering covariates, including sequence context and replication timing.

### Integrative data analysis

Subtypes were identified on the basis of individual types of data (mRNA sequencing data, whole proteome, phosphoproteome) expressed in at least 70% of patient samples. Median absolute deviations (MADs) were calculated for the individual data, and the top 10% of the most variably expressed genes were subjected to subsequent statistical analyses. On the basis of the MAD values, we then selected the molecules with the top 10% of MADs: 1,468 genes, 723 proteins, and 128 phosphopeptides. Next, we applied network integration based on consensus clustering to conduct unsupervised classification and to identify proteogenomic features that represent characteristic expression patterns for each cluster. The three data types were aggregated to generate an integrated network and similarity matrix, followed by the designation of consensus clusters. The criteria for determining the number of clusters were as follows: relatively high consistency within clusters, a relatively low variation coefficient, and no appreciable rise in the area under the cumulative distribution function (CDF) curve. The samples most representative of the clusters were identified on the basis of their positive silhouette width.

To determine the representation of intrinsic subtypes of our cohort, the PAM50 model was applied to TPM-normalized RNA-seq data using the “genefu” R package (v3.12)^35^.

Single-sample gene set enrichment analysis (ssGSEA) was performed for each sample using log2-transformed and quantile-normalized RNA-seq data and the global proteome for the molecular signatures database (MSigDB) GeneSets^36^ via GenePattern 2.0^37^ and Gene Ontology (GO) biological process signatures. The gene set scores represent the enrichment scores obtained from ssGSEA. To generate the receiver operating characteristic (ROC) curve and calculate the area under the curve (AUC) from the ssGSEA results and binary phenotype classification, we used ssGSEA_ROC from GenePattern 2.0. Statistical significance was determined using a false discovery rate (FDR) of less than 0.01. To obtain individual enrichment scores for each pairing of a sample and a gene set, we used the ssGSEA projection on the GenePattern server using t test statistics with a *p-*value less than 0.001.

### Immune profiling and downstream analysis

To calculate RNA-based tumor immune scores and estimate immune-cell-specific contributions to each tumor, TPM data were analyzed using ESTIMATE^38^, CIBERSORT in absolute mode^39^, xCell^40^, and the MCP counter^41^. We also inferred the immune cell infiltration by ssGSEA using a published immune gene signature^42^. Protein-based immune scores for stimulatory and inhibitory immune modulators, as well as the set of HLA proteins, were calculated as the mean of the protein log ratios in each set defined in Thorsson et al^43^.

### Kinase phosphorylation outliers

To identify the kinase activity characteristic of each PAM50 and major integrative clusters (iClusters), we used BlackSheep’s differential extremes value analysis module^44^. For each phosphosite, the median and interquartile range (IQR) were calculated across all tumors. A site was defined as an outlier if it was more than 1.5 times the IQR above the median. The phosphosites were then collapsed into proteins by counting outlier and nonoutlier values per sample. For each group of interest (e.g., PAM50 or iCluster), proteins that were not enriched in outliers in that group and proteins without at least 30% of samples with an outlier were removed. Following filtering, outlier and nonoutlier sites per gene were counted for each group of interest, and Fisher’s exact test was used to calculate a *p*-value with multiple hypothesis correction via the Benjamini‒Hochberg (BH) procedure. For additional insight into kinase activity, we visualized the enrichment of kinase activation loop phosphorylation, which was calculated using a rank sum test.

### Kinase-substrate enrichment analysis (KSEA)

KSEA was performed using the KSEA App web-based tool^45^ with phosphosite data with a cutoff of p < 0.05 and a substrate count of ≥ 1. Student’s *t*-test was used to calculate the p value, and the mean ratio was used to determine the fold change compared with the other four subsets. KSEA computes a normalized score to measure relative kinase activity in cluster 1 versus others using the difference in the mean log fold changes of predefined substrate groups of the kinase, as referenced from the PhosphoSitePlus^46^ and NetworKIN databases^47^. In the latter database, kinase–substrate annotations with a NetworKIN score of less than five are discarded. The mean log fold changes of all substrates were divided by the standard deviation of log fold changes across all phosphosites, followed by an adjustment to the score for the number of substrates in the data that were annotated to the kinase. The statistical significance of the scores was calculated using a one-tailed z test, followed by BH multiple testing correction. Kinases with z scores and corresponding p values less than 0.05 were considered significant.

## Results

### Patient Cohort Description

The baseline characteristics of the 126 patients with YBC are summarized in Supplementary Table 1. The median patient age was 37 years (range: 25–40). Seventy-six patients (60.3%) had stage II disease, and 64 patients (50.8%) had node metastasis at the time of diagnosis. Sixty-five patients (51.6%) had a histological grade of 3. ER was positive in 84 patients (66.7%), PR was positive in 73 patients (57.9%), and HER2 was positive in 25 patients (19.8%). Based on the clinical classification of breast cancer with ER, PR, HER2, and Ki-67^48^ statuses, 33 patients were classified as luminal A type (26.2%), 55 as luminal B type (43.7%), 10 as HER2 type (7.9%), and 25 as TNBC type (19.8%). All patients were diagnosed with invasive ductal carcinoma. Overall, 113 patients (89.7%) underwent systemic chemotherapy, 104 patients (82.5%) underwent adjuvant radiotherapy, and 89 patients (70.6%) underwent adjuvant hormone therapy. The median follow-up period was 94.3 months (range: 4.0–254.3), 37 patients (29.4%) experienced recurrence, and 17 (13.5%) died.

All of the patients in this study (*n* = 126, 100%) were initially diagnosed under the age of 40 and premenopausal, and TCGA data largely consists of patients with breast cancer over the age of 40 (*n* = 1,998, 91.1%). Compared with the TCGA cohort (*n* = 1116), our patients demonstrated a greater proportion of the basal subtype (28.6% KNCC-YBC vs. 16.5% TCGA) and HER2-enriched subtype (15.9% vs. 6.3%). This difference was maintained when compared to young patients (age ≤ 40) in the TCGA cohort (basal type 17.7%, HER2 type 4.4% in TCGA_YBC [*n* = 158]). This finding is comparable to the findings of a previous study in which Asian patients with breast cancer had a greater prevalence of HER2-positive cancers than Caucasian women did^10,49^.

### Genomic landscape of early-onset breast cancer

**Fig. 1:**
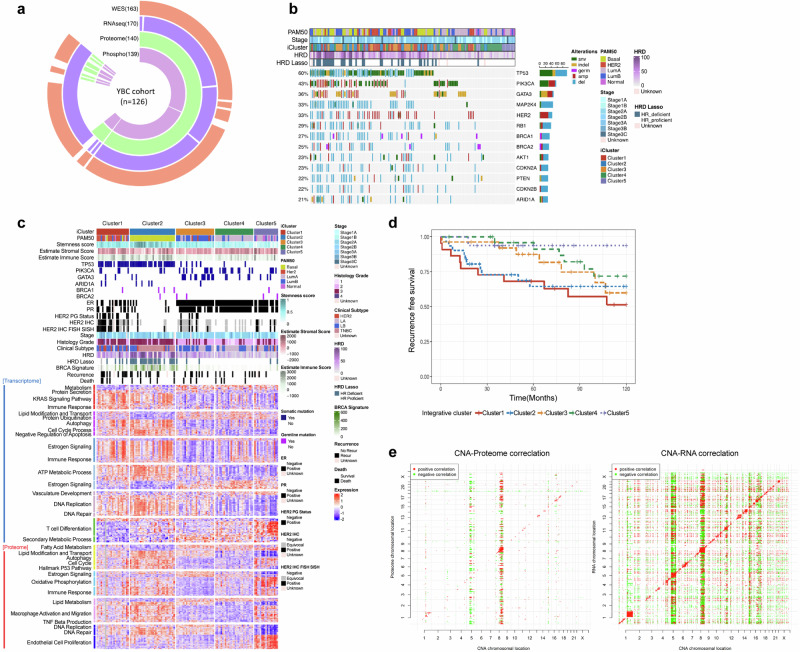
Molecular portraits of early-onset breast cancer. **a** Data structure outlining the molecular profiles of 126 patients with early-onset breast cancer, categorized according to each type of molecular data generated in the current study. WES (*n* = 163), RNA-seq (*n* = 170), proteome (*n* = 140), and phosphoproteome (*n* = 139) data were generated for 178 young- breast cancer patients. Among these patients, multiomic data were generated for 126 individuals. **b** Genomic landscape of major breast cancer driver genes on the basis of mutational and somatic copy number status. Genomic alterations are color-coded in accordance with the type of mutation. The bar graph on the right denotes the total count of each specific genomic alteration observed. PAM50: PAM50 breast cancer subtypes; Stage: Pathological stage of breast cancer; iCluster: 5 integrative clusters based on proteogenomic analysis of the current study cohort; HRD: the level of homologous recombination deficiency on the basis of next-generation sequencing data; HRD LASSO: classification of HRD status from next-generation sequencing data; **c** Heatmap illustrating supervised clustering of differentially expressed genes (upper panel) and proteins (lower panel) across integrative molecular clusters (Kruskal‒Wallis test, FDR *p* < 5 × 10^−5^). The pathways enriched by the differentially expressed genes and proteins are annotated on the left. **d** Kaplan‒Meier curves showing the progression-free survival outcomes of patients in the integrative clusters in the dataset. Stratification of patients on the basis of comprehensive multiomics data yielded five distinct molecular clusters, each associated with a different clinical outcome. Clusters 1 (red), 2 (blue), 3 (yellow), 4 (green), and 5 (violet) are shown. **e** Correlations of somatic copy number alterations (SCNAs, x-axis) with mRNA (left) and protein (right) abundances (y-axis).

We performed an integrated analysis of molecular data from patients through WES, WTS, and mass spectrometry-based proteomics, which provided robust quantification of proteins and phosphoproteins (Fig. 1a). Whole-exome sequencing of 126 samples revealed 30,193 somatic mutations in the study cohort. Each patient harbored a median of 119 somatic mutations per sample (range: 2–2858 somatic mutations). Significance analysis of the somatic mutations, employing covariates related to the mutational processes, identified the key mutated genes, including *TP53, PIK3CA, GATA3, MAP2K4*, and *ARID1A* (Fig. 1b). *TP53* (60%), *PIK3CA* (43%), and *GATA3* (36%) were the most frequently mutated genes in this cohort. GATA3 somatic mutations were mostly mutually exclusive with TP53 mutations (Fisher’s exact test, *P* < 0.01), with *PIK3CA* mutations exhibiting similar mutual exclusivity patterns (*P* < 0.01). Overall, approximately 68% of the patients possessed at least one mutation in one of these three genes. The prevalence of somatic mutations in *MAP2K4* and *ARID1A* in breast cancer has also been documented in other studies^50–53^, indicating the presence of a congruent mutational profile within the early-onset Asian breast cancer demographic.

### Molecular subtype classification on the basis of multiomic profiles

In addition to classical pathological and clinical laboratory-based stratification of the molecular subtypes of the cohorts using hormone receptor and HER2 immunohistochemistry (IHC) analyses, we attempted a robust molecular classification of YBC cohorts based on a multiomics dataset. Unsupervised clustering of the three data platforms (proteome, transcriptome, and phosphoproteome) resulted in discordant stratification unique to each molecular layer. A consensus integrative classification encompassing the three molecular data layers produced five iClusters within the young Korean breast cancer cohort (Fig. 1c).

Cluster 1 was significantly concordant with the classical PAM50-based HER2-enriched subtype. This cluster was characterized by a high prevalence of *HER2* amplification and *TP53* mutations (69.6% and 82.6% in cluster 1, respectively). The key pathways in this cluster included protein secretion, DNA replication, and repair. Cluster 2 was almost invariably consistent with the basal-like cancer type and presented a high frequency of *TP53* mutations and an absence of hormone receptor and HER2 mutations. Cluster 2 was associated with the activation of pathways such as KRAS signaling, autophagy, and cell cycle processes and the negative regulation of apoptosis. Clusters 1 and 2 demonstrated the worst prognosis, with recurrence and metastasis events frequently observed within 5 years after surgical resection (Fig. 1d). Cluster 3 largely overlapped with the classical luminal B subtype, whereas cluster 4 demonstrated closer alignment with the classical luminal A subtype. The important pathways of cluster 3 and cluster 4 included estrogen signaling, the cell cycle, DNA replication and repair, and fatty acid metabolism (Fig. 1c). Those clusters of young Korean patients with breast cancer experienced more frequent recurrence and/or death events 5 or more years post-diagnosis of breast cancer (Fig. 1d). All cases of late relapse in these clusters were ipsilateral (11/11, *P* < 0.00001, Fisher’s exact test); thus, the late recurrent tumors seemed to have originated from residual and/or resistant tumor cells left from their primary tumors after a sustained period of dormancy rather than from the manifestation of a second primary tumor. In contrast, basal-type and HER2-type Korean YBC demonstrated completely opposite biphasic patterns of survival probability; these groups’ relapse and consequent death events were more clustered in the first 5 years, followed by a plateau in survival probability. The dichotomy of biphasic survival probability between luminal and nonluminal Korean patients with breast cancer is substantially distinct from the clinical course of Caucasian breast cancer, where luminal A breast cancer shows the most indolent phenotype, followed by luminal B breast cancer and HER2 breast cancer, with the basal group having the highest risk patterns^54^. The results of the integrated analysis revealed a distinct subtype, cluster 5, which had a very good prognosis, unlike the other four clusters (Fig. 1d). The significant pathways of cluster 5 included vasculature development, endothelial cell proliferation, and oxidative phosphorylation.

To examine the impact of somatic copy number alterations (SCNAs) on the levels of mRNAs and proteins, we conducted univariate correlation analysis to identify features that exhibited statistically significant correlations (Fig. 1e). This analysis considered changes in ‘cis’—affecting genes within the altered loci—and ‘trans’—impacting genes located elsewhere. We evaluated the genes in this cohort, each with corresponding SCNA, mRNA, and protein data, by calculating the correlation and its statistical significance for all CNA–mRNA and CNA–protein pairings. Notably, 61% of the CNA-mRNA pairs and 30% of the CNA–protein pairs demonstrated significant positive ‘cis’ correlations. We further scrutinized the subset of these pairs that included well-characterized oncogenes and tumor suppressor genes. ‘Trans’ effects appeared as prominent vertical bands in the analysis, with frequency histograms highlighting significant ‘hot spots’ of ‘trans’ correlation. Interestingly, correlations between CNA and proteins were generally weaker than those observed for CNA and mRNAs. The most pronounced ‘trans’ relationships at the protein level were identified in specific CNA regions, including regions of chromosome 5 and chromosome 8, in early-onset Korean breast cancer.

### Targetable elements associated with clusters and genomic alterations

**Fig. 2:**
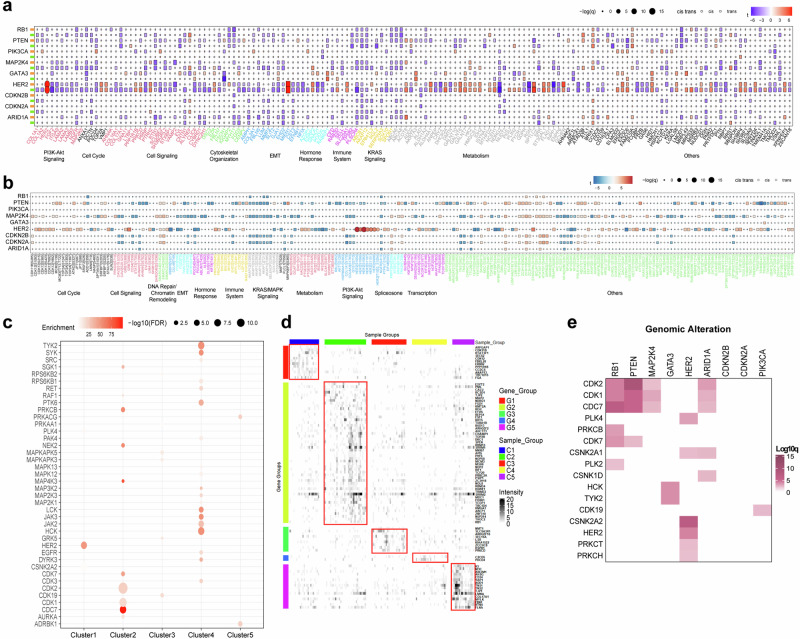
Targetable elements associated with clusters and genomic alterations from proteogenomic analysis of early-onset East Asian breast cancer. *Cis-* and *trans-*effects of major genomic alterations, including mutations and copy number variations, on protein (**a**) and phosphoprotein (**b**) levels. The *cis* and *trans* effects of each genomic alteration can be categorized into major cancer hallmark pathways. **c** Plot depicting kinases that exhibited preferential substrate phosphorylation within each integrated molecular cluster. **d** Heatmap showing the fraction of outlier values in each sample per protein. The proteins shown are kinases highly phosphorylated in each cluster, with an FDR of less than 0.01 according to BlackSheep. The top panel shows the classifier based on the five major integrative clusters (iClusters). The right panels depict the abundance of the kinase activation loop and kinase substrate enrichment. **e** Heatmap showing q values from kinase substrate enrichment analysis for enrichment of phosphorylation outliers (y-axis) in samples with the indicated mutated gene (x-axis). Kinases with an FDR of less than 0.01 are shown.

Next, we examined the functional impact of genetic alterations on the abundance of both *cis*-acting (cognate gene products) and *trans-*acting (other gene products) proteins and their phosphorylation sites (Fig. 2a, b). Several *cis* and *trans* associations were discernible. These include enhanced expression of HER2 proteins (Fig. 2a) and strong *cis*-phosphorylation (Fig. 2b) in HER2-positive breast cancers, cementing their role as potent drivers of alterations in a subset of breast cancers. Migration and invasion enhancer I (*MIEN1*) and growth factor receptor-bound protein 7 (*GRB7*) are genes located proximal to the *ERBB2* gene and are concomitantly overexpressed in HER2-positive breast cancer subgroups (Fig. 2a, b). *MIEN1* has been shown to functionally increase the invasive and migratory phenotypes of various types of cells in breast cancer^55^. Additionally, GRB7 overexpression facilitates the phosphorylation of both AKT and HER2/neu in HER2/neu-overexpressing cells, promoting tumor growth in vivo^56^. These findings suggest additional roles for these proximal genes in breast cancer. Several phosphorylation sites associated with common breast cancer oncogenes were identified and clustered in the cell cycle and DNA repair activities, which is consistent with their established roles in cancer.

We investigated which kinase activities were enriched in the molecular subgroups defined in this study (Fig. 2c). As expected, HER2 kinase activity was upregulated in cluster 1, which was highly correlated with the classical PAM50 Her2 subgroup. Cluster 2 (basal subgroup) was enriched with cell cycle kinases, including CDC7, CDK1, and CDK2. Hormone receptor-positive cancer groups (clusters 3-4) presented increased activity of kinases involved in immune regulation. To identify potential therapeutic targets specific to each iCluster subtype, we used phosphoproteomic data to determine kinase activation (Fig. 2d). We identified phosphorylated kinases that were enriched in each iCluster subtype via outlier enrichment analysis. The dataset revealed several druggable targets and kinases, including ERBB2 (trastuzumab), in the cluster 1 subtype. The cluster 2 subtype includes isolated PML (tretinoin) and RCSD1 (imatinib). PDCD4 (paclitaxel) was found in cluster 4, whereas CD34 (prednisolone) and TJP1 (dexamethasone) were present in cluster 5.

These proteins and kinases are potential targets for subtype-specific treatment. We also investigated the associations between specific genomic alterations and kinase activity (Fig. 2e). We identified 16 kinases significantly associated with genomic alterations. *RB1, PTEN*, and *MAP2K4* mutations formed a strong subcluster with increased activity of several canonical cell cycle kinases, including the CDK1, CDK2, CDK7, and CDC7 proteins. These associations were also detected in *ARID1A-*mutant breast cancers. The PLK4, CSNK2A2, HER2, PRKCT, and PRKCH proteins are associated with HER2 genomic alterations. GATA3 genomic alteration was associated with increased activity of the HCK and TYK2 kinases, whereas PIK3CA genomic alteration was linked with the CDK19 kinase.

### Proteogenomic analysis of HER2-type YBC

**Fig. 3:**
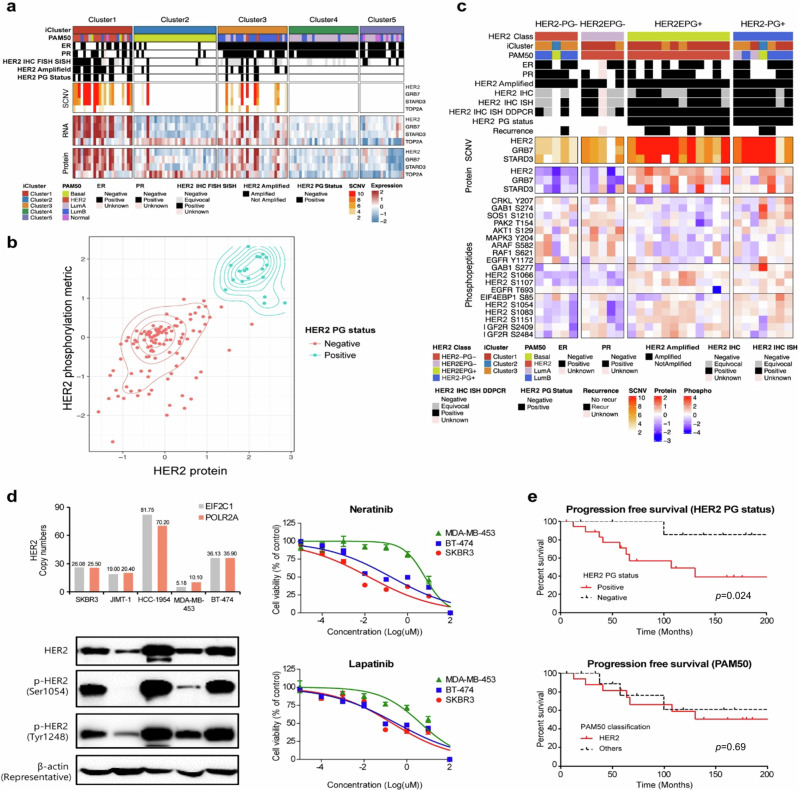
Proteogenomic classification of HER2 status in YBC. **a** Proteogenomic analysis of the HER2 locus in the current cohort. The heatmap displays the clinical and molecular data (top panel), SCNA data (center upper panel), RNA expression data (center lower panel), and protein expression data (bottom panel) of genes located near HER2 on chromosome 17q in the corresponding samples. HER2 IHC FISH SISH: pathological index of HER2 status from immunohistochemistry, fluorescence in situ hybridization or silver-enhanced in situ hybridization, HER2 PG Status: Her2 proteogenomic status. **b** Proteogenomic classification of the HER2 breast cancer subtype. Proteogenomic status of HER2 combined with HER2 protein levels (x-axis) and HER2 phosphorylation status (y-axis). HER2 proteogenomic status was stratified and depicted as either blue (positive) or red (negative). **c** Phosphopeptide levels of components of the HER2 signaling pathway according to the refined classification of HER2. The top panel of the heatmap outlines the subtype classifications and clinical marker status for each sample, whereas the center panels denote SCNAs and protein levels for genes in the amplicon closely associated with HER2, followed by the corresponding protein levels. The bottom panel illustrates the abundances of phosphopeptides, such as serine residues 1066, 1107, 1054, 1083, and 1151 from the HER2 pathway. **d** HER2 protein levels and drug response to HER2 inhibitors in breast cancer cell lines. (Left upper) Distribution of HER2 copy number in HER2-positive cell lines. The HER2 copy number was measured in HER2-positive breast cancer cell lines via droplet-digital PCR (ddPCR). EIF2C1 and POLR2A were used as reference genes to determine the ratios of HER2 to EIF2C1 or POLR2A. (Left lower) Western blot analysis was conducted to assess the expression and phosphorylation status of HER2 in HER2-positive breast cancer cell lines. The HER2-positive breast cancer cell lines SKBR3, JIMT-1, HCC-1954, MDA-MB-453, and BT-474 were separated via SDS‒PAGE and immunoblotted. Western blotting was performed for total HER2, p-HER2 (Ser1054, Tyr1248), and β-actin. (Right) Drug sensitivity test of HER2 inhibitors (neratinib, lapatinib) in breast cancer cells phosphorylated at serine 1054. MDA-MB-453, BT-474, and SKBR3 cells were treated with neratinib or lapatinib for 48 h. Data are presented as the mean ± SEM. Drug sensitivity assays were performed independently in triplicate. **e** Kaplan‒Meier curves showing progression-free survival outcomes according to HER2 PG status or PAM50 class.

We assessed the HER2 driver status by integrating genomic and proteomic data to refine its classification and clinical significance. HER2 status was also grouped by classical IHC and in situ hybridization testing following ASCO-CAP guidelines (HER2+: IHC score of 3+ or an IHC score of 2+ fluorescence in situ hybridization (FISH); silver-enhanced in situ hybridization (SISH): HER2 equivocal status: IHC score of 2+ without FISH results or amplification by FISH without IHC results)^57^. Proteogenomic analysis revealed that 19 patients who presented high levels of both HER2 protein and phosphorylated HER2 were designated as HER2 proteogenomic status positive (HER2 PG+) (Fig. 3a). HER2 PG+ cases were enriched mostly in cluster 1, followed by cluster 3. The majority of HER2 PG+ samples (16 out of 19) demonstrated largely consistent pathological designations with confirmed HER2-positive expression according to IHC, FISH, and SISH tests. The remaining three patients who were grouped according to HER2 equivocal status demonstrated a high *HER2* copy number as a result of droplet digital PCR analysis. Although we identified 28 patients with *HER2* gene amplification on the basis of copy number alterations, only 19 of these patients were positive for HER2 by PGstatus. Consistent with the proximal location of the *HER2* gene on chromosome 17q12, the copy number, RNA, and protein expression of GRB7 and STARD3 were concomitantly increased in patients with HER2 amplification (Fig. 3a). The protein levels and phosphorylation metrics of HER2 more robustly and clearly segregated the designation between patients with positive (HER2-PG+) and negative (HER2-PG-) HER2 PG status (Fig. 3b).

In the stratification of patients with breast cancer with *HER2* driver dependency, there was a notable incompatibility between conventional PAM50-based classification, classical pathological HER2 type designation, and proteogenomic-based integrative clustering. To gain deeper insights into the biological characteristics that led to sample clustering within the HER2-enriched subtype (HER2E) group despite inconsistent ERBB2 status, we conducted an analysis of phosphosites that are known to canonically relay kinase signaling from the human ERBB2 protein. Patients assigned to the HER2E group were divided into HER2EPG- and HER2EPG+ groups on the basis of their PG status (Fig. 3c). As anticipated, all PAM50 HER2EPG+ samples presented elevated levels of ERBB2 phosphopeptides. Five additional cases of the PAM50 HER2-enriched subtype were classified into the HER2EPG- subgroup. A close examination of the five cases revealed that these samples shared common activated receptor tyrosine kinase (RTK) downstream pathway signatures, including ARAF, MAPK3, and AKT1 (Fig. 3c). This finding indicates that the PAM50 simplification scheme mistakenly designates some samples with activated RTKs as belonging to the HER2 subtype, regardless of their subtype-specific nature. Indeed, some samples demonstrated an activated phosphorylation profile of IGF2R, further suggesting that some breast cancer samples might be targetable with precise segregation of RTKs with a suitable driver dependency nature^14^.

Quantitative phosphoproteomic analysis revealed that HER2 PG+ cases exhibited elevated levels of HER2 serine phosphopeptides, including serine residues 1054 and 1066, in comparison to PG- cases (Fig. 3c). Given the close association between the phosphorylation status at serine 1054 (S-1054) and HER2 PG status, we investigated its potential as a predictive biomarker for the sensitivity of breast cancer cell lines to HER2 inhibitors. Despite the limited number of cell lines analyzed, the phosphorylation of S-1054 was found to be correlated with the response to lapatinib and neratinib treatments (Fig. 3d). We then compared the effects of a HER2 inhibitor on the phosphorylation status of HER2-positive breast cancer cell lines. The phosphorylation level of HER2 at tyrosine 1248 (Y-1248) decreased following treatment with neratinib. The phosphorylation of S-1054 decreased in HER2-positive breast cancer cell lines following neratinib treatment (Supplementary Fig. 1). These results suggest that decreased phosphorylation of S-1054 on HER2 could serve as a predictive marker for sensitivity to HER2 inhibitors.

Among patients with amplified HER2, the proteogenomic status of HER2 was potentially prognostic, whereas PAM50 HER2 enrichment was not (Fig. 3e). The patients with HER2 PG+ status tended to have poorer clinical outcomes among the patients with amplified HER2 (*p* = 0.024).

### Proteogenomic analysis of the tumor immune microenvironment

**Fig. 4:**
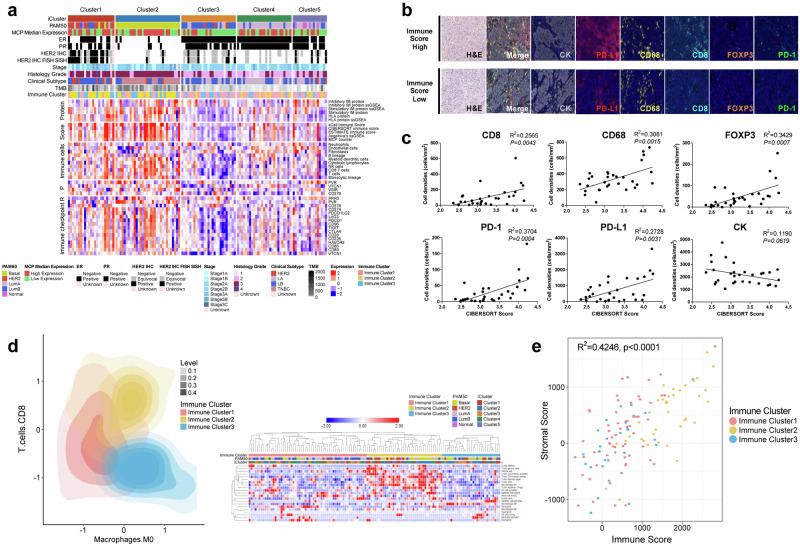
Immunological landscape of early-onset Korean breast cancer. **a** Heatmap showing the wide range of expression levels for immune-related features in each integrative molecular cluster. Protein-derived signatures for immune modulator gene sets are depicted in the top panel. Z scores of RNA-based immune signatures from xCell, CIBERSORT, ESTIMATE, gene sets from Angelova, and the MCP counter are shown in the second data panel. The third to fifth data panels show log2 ratios for normalized RNA-seq and proteomics data (the phosphoprotein is the median for all sites on a given protein) for immune cells and immune checkpoint targets, such as CD276, TIGIT, LAG3, and CTLA4. MCP median expression: ER: pathological staining of estrogen receptor, PR: pathological staining of progesterone receptor. **b** Multiplex imaging portrays the immune microenvironment of the tumor. The upper and lower panels represent samples that correspond to high and low computational immune scores, respectively. The samples were probed for CK (cytokeratin), PD-L1, CD68, CD8, FOXP-3, and PD-1. The morphological features are indicated by H&E staining. **c** Graphs depict the linear regression for correlations between cell counts per total area (cells/mm^2^) of each patient from all available ROIs for multiplex imaging probes and the CIBERSORT absolute immune score. *P < *0.05* **d** YBC samples are classified into immune-hot (yellow), immune-intermediate (pink), and immune-cold (blue) groups. The heatmap on the right illustrates various immune components (y-axis) as per the immune clusters. **e** Collinearity between immune and stromal scores in early-onset breast cancer. The scatter plot shows the relationship between the computational immune score (x-axis) and the stromal score (y-axis), with colors corresponding to the three different immune clusters. Spearman rank correlation coefficient analysis of the immune score (x-axis) and stromal score (y-axis) for immune clusters 1 ~ 3 (R^2^ = 0.4246, *p* < 0.0001).

Using transcriptome, proteome, and phosphoproteome datasets, we attempted to classify patients according to their immune signals to understand their tumor immune microenvironments at the molecular and cellular levels (Fig. 4a). Clusters 1 and 2 were enriched in immune signatures, and the mRNA and protein expression levels of immune checkpoint targets, such as CD276, TIGIT, LAG3, and CTLA4, were also elevated relative to those in the other clusters. Cluster 3 included an increased proportion of patients with fewer immunogenic features, with decreased numbers of T, B, NK, and monocytic lineages, as well as immune checkpoint targets. Multiplex IHC staining of five different immune cell markers (CD8, CD68, FOXP3, PD1, and PD-L1) confirmed the validity of inferring tumor immune microenvironment features from a multiomics dataset (Fig. 4b). Correlation analyses of multiplexed IHC staining data revealed that YBC immune cells have highly intercorrelated molecular features, confirming the established correlative nature of immune checkpoint molecules, such as augmented PD-L1 staining, which is highly correlated with an increased number of exhausted CD8+ Tregs and immunosuppressive macrophages in the tumor parenchyma (Supplementary Fig. 2). CD8-positive T-cell IHC further validated the inferred active immunogenic tumor microenvironment (Fig. 4c). We also identified three immune-based subtypes with distinct immune characteristics (Fig. 4d). Among the three clusters, immune-cluster 2 was assigned an immune hot phenotype, immune-cluster 3 an immune cold phenotype, and immune-cluster 1 an immune -intermediate phenotype. There was a positive correlation between the stromal and immune scores according to the three immune-based subtypes (R^2^ = 0.4246, *p* < 0.0001; Fig. 4e).

Classical RNA-seq-based PAM50 classification demonstrated that each subtype of YBC comprises a subset of patients with an active immune signature (Supplementary Fig. 3), albeit with higher immune scores in the basal PAM50 subtype (Supplementary Fig. 4) (ANOVA, *P* = 0.007). The active immune tumor microenvironment consisted of elevated levels of stimulatory immune proteins and HLA proteins and increased proportions of tumor-infiltrating lymphocytes (TILs) and myeloid dendritic cells (Supplementary Fig. 3).

In contrast to the PAM50 stratification, integrative multiomics-based clustering generated immune microenvironment features that segregated cluster 3 from the other clusters (ANOVA, *P* = 0.001) (Supplementary Fig. 5). The evaluation of the clinical characteristics of the poorly immunogenic subgroup tended to be associated with a poor clinical prognosis (Supplementary Fig. 6). The clinical association of poor immunogenic features with a poor prognosis was observed in the luminal B PAM50 subtypes in this study cohort (Fig. 4a), suggesting that immune microenvironment features in specific subgroups of early-onset breast cancer identified through multiomics integration may serve as prognostic molecular markers for stratifying high-risk late-onset luminal B subtype patients.

### Proteogenomic analysis of homologous recombination-deficient (HRD) breast cancer

**Fig. 5:**
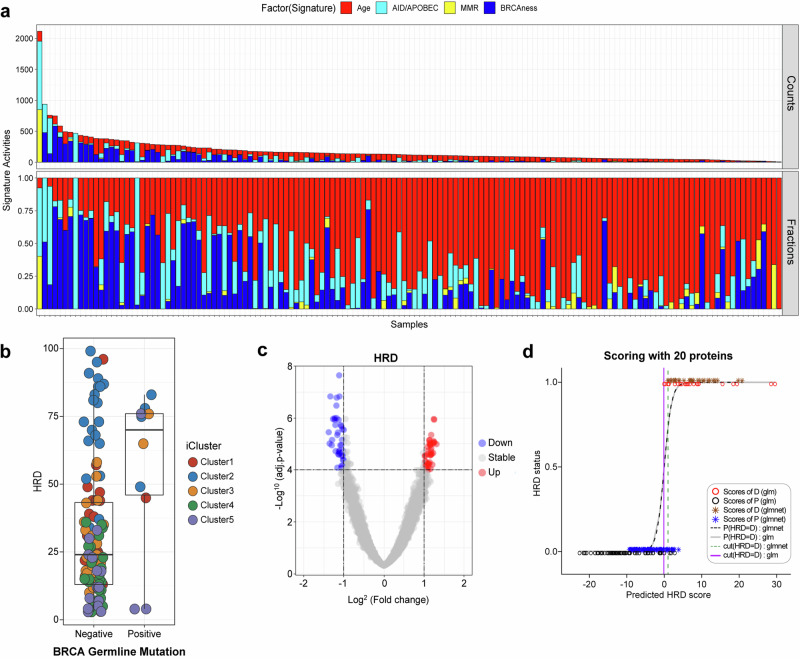
Proteogenomic analysis of homologous recombination-deficient YBC. **a** Mutational signature in early-onset breast cancer displaying the quantity (upper) and proportion (lower) of somatic mutations per sample belonging to each mutational signature. These include aging (red), APOBEC (cyan blue), mismatch repair defects (MMR, yellow), and BRCAness (blue) signatures. **b** Relationship between the homologous recombination defect (HRD) index and BRCA germline mutations. Germline BRCA mutations have a limited correlation with the degree of HRD. **c** Identification of protein elements that are highly correlated with the degree of HRD in breast cancer samples. **d** Predictive accuracy of different scoring methods in forecasting HRD status on the basis of scores derived from 20 proteins associated with HRDness. The data are presented using two different modeling techniques: a generalized linear model and elastic net regularization. The x-axis represents the predicted HRD score values for each sample. The y-axis indicates HRD status, with 0 representing non-HRD (HRP) and 1 representing HRD. Scores of D, P (glm): scores derived from the generalized linear model for Dataset D or P, represented by red and black circles. Scores of D, P (glmnet): scores derived from the elastic net model for Dataset D or P, represented by brown and blue asterisks. P (HRD = D): glm/glmnet: predicted logistic probability of HRD using the glm or glmnet model, depicted by a gray solid line or black dashed line. Threshold (HRD = D): glm/glmnet: The threshold value for HRD is D using the glm or glmnet model, shown as a solid or dotted line estimated from the maximum ROC. The thresholds help distinguish between HRD and non-HRD samples, providing a visual comparison of the predictive power and accuracy of the glm and glmnet models.

To investigate the mutational processes involved in early-onset East Asian breast cancer, we deconvoluted the mutational signatures in the cohort. Four prevalent mutational signatures—BRCAness, APOBEC, mismatch repair defects, and aging signatures—were observed (Fig. 5a). We also examined germline pathogenic variations, defined as mutations that were reported as the most likely disease-causing variants in ClinVar or mutations that are truncating proteins in 10 genes reported to increase breast cancer susceptibility with a relatively strong penetrance^10,58,59^. Among the 10 breast cancer susceptibility genes, 10.3% (13/126) of patients harbored pathogenic germline variations, whereas one patient was observed to possess pathogenic variations in both RAD50 and BRCA1. Pathogenic *BRCA1/2* germline mutations were more prevalent in Korean patients with YBC, affecting 8.7% of patients in the study cohort compared with 3.8% of patients in the TCGA database (Fisher’s test, *P* < 0.02). BRCA germline mutations, which are classical biomarkers of BRCAness and PARP inhibitors, only partially explained the proportion of patients with elevated genomic HRD levels (Fig. 5b). We interrogated the proteome data to search for proteins closely associated with the features of HRD (Fig. 5c). Among these, 20 highly ranked proteins were chosen whose combination could segregate genomic HRD-positive and HRD-negative cancers (Fig. 5d).

### Prognostic significance of protein‒RNA correlations

Positive correlations existed between the majority of the quantified proteins (>90%) and their corresponding mRNA transcripts within the present study cohort, with a median of 0.34 (Supplementary Fig. 7a). These correlations were not contingent upon technical factors such as the average protein precursor area or the number of peptide spectral matches. Structural ribosomal proteins and those involved in translational processes presented the lowest correlations between protein and transcript abundance (Supplementary Fig. 7a), highlighting their function and regulatory control, primarily at the protein level.

Notably, proteins derived from transcripts identified in breast cancer drivers, including estrogen receptor responses, exhibited the strongest mRNA‒protein correlations (Supplementary Fig. 7a), underscoring a direct connection to the protein phenotype in the major canonical breast cancer driver signaling pathway. In hormone receptor-positive breast cancer patients, the subgroup with a high RNA‒protein correlation was associated with a poorer prognosis than the subgroup with a low RNA‒protein correlation (Supplementary Fig. 7b, *p* = 0.0049). This is reinforced by the greater correlation of genes that respond to estrogen receptor signaling blockade (Supplementary Fig. 7a). These findings suggest that protein‒RNA correlations could serve as promising prognostic markers for predicting late recurrence in the luminal subtype, necessitating comprehensive external validation accounting for potential confounders in future investigations.

## Discussion

Patients with YBC have a poor prognosis not only in Western countries^7,60^ but also in Korea^5^, particularly those with luminal A and luminal B/HER2-type breast cancer^61^. This phenomenon has been attributed to tamoxifen resistance^5,61^. Our proteogenomic analysis stratified luminal breast cancer patients into a YBC group with late recurrence (clusters 3 and 4) and very good survival (cluster 5). This classification system can be used to identify patients requiring long-term endocrine therapy.

According to the genomic expression analysis, YBC had significantly lower ERα, ERβ, and PR mRNA expression but higher HER-2 and EGFR expression. In addition, lower ERα and higher EGFR mRNA expression were significant predictors of inferior disease-free survival^62^.

In our Korean YBC cohort, *TP53, PIK3CA* and *GATA3* were the most frequently mutated genes, which is consistent with the results of the TCGA cohort. The TCGA cohort provides three age groups (≤45, 46-69, and ≥70), and PIK3CA and TP53 were the most common somatic mutations in all three age groups while GATA3 was the third most common and independent somatic mutation in young patients, affecting 15.2%^63^.

Proteogenomic analysis integrating the phosphorylation status of HER2 can be used to identify true HER2+ patients. Traditionally, HER2 has been tested for gene amplification or protein overexpression by IHC and ISH. Through proteomic profiling, not only quantitative expression but also posttranslational modification of HER2 could be used for a more precise classification. We identified the proteogenomic status of HER2 as a potential prognostic marker among patients with amplified HER2, as patients with HER2 PG+ status tended to have poorer clinical outcomes than PG- patients. Especially for Asian patients with breast cancer who show a high proportion of HER2 enrichment, HER2 PG status may help predict the clinical outcomes of HER2-targeted therapies. Furthermore, compared with PG- patients, HER2 PG+ patients presented increased phosphorylation of the HER2 protein. Phosphorylation of the HER2 protein has been extensively studied as an essential regulatory mechanism for protein function, but relatively little is known about the effects of serine residues compared with those of tyrosine residues. Our results suggest that the phosphorylation status of the serine 1054 residue is a good marker for estimating the PG status of HER2.

PAM50 is a classification tool used to characterize and predict the outcomes of patients with breast cancer^64–66^. However, our results demonstrated that the proteogenomic status of HER2 was a potential prognostic factor, whereas PAM50 HER2 enrichment was not. Patients with a HER2 PG+ status tended to have poorer clinical outcomes. The HER2-positive subtypes show ethnic disparities, and Korean patients with breast cancer have a high degree of HER2 enrichment^10,49,67^. Our results suggest that HER2 PG status is required to select true-HER2+ patients who respond well to anti-HER2 treatment. As expected, all of the PAM50 HER2EPG+ samples had high levels of HER2 phosphopeptides, whereas the PAM50 HER2EPG- samples had markedly lower levels but elevated levels of phosphorylation of other ERBB family members and of the mitogen-activated protein kinase (MAPK) signaling pathway compared with PG+ samples. These findings suggest that complementary pathways that can replace HER2 signaling may be targetable in PAM50 HER2E tumors without HER2 amplification.

The tumor microenvironment (TME) is a critical factor that influences the response to immunotherapy. The cytotoxic subset of CD8+ T cells can eliminate cancer cells and is associated with improved patient survival. Conversely, immunosuppressive regulatory CD4+ T cells (Tregs) or macrophages are associated with a poorer prognosis^68^. “Hot” tumors characterized by high levels of T-cell infiltration generally exhibit more favorable responses to immunotherapies than “cold” tumors, which have limited T-cell infiltration^69^. The existence of TILs within the TME indicates the presence of an inherent immune response against the tumor, which is linked to a more favorable prognosis and increased responsiveness to chemotherapy^70^.

Compared with more immunogenic cancers, breast cancer, which is traditionally considered to be poorly immunogenic, or “cold” with a modest tumor mutation burden (TMB) and a low TIL count, shows a notable prevalence of APOBEC mutational signatures^71–73^. The mutational load in BC exhibits significant variability, and tumors with higher TMB may exhibit a more favorable response to immune checkpoint inhibitors (ICIs)^74^. Although PD-L1 expression is commonly used as a biomarker, it alone is not sufficient as a predictor, demonstrating its predictive value mainly in metastatic TNBC. TNBC and HER2-positive BC are considered more immunogenic, whereas hormone receptor-positive BC is considered less immunogenic. However, certain patients with HER2-positive or hormone receptor-positive BC may also show promise for immunotherapy in addition to TNBC^75^.

The study cohort revealed a clinical association between an unfavorable prognosis and poor immunogenic characteristics, specifically within the luminal B PAM50 subtypes. This implies that, in cases where hot immune groups are identified within specific subtypes, it is essential to consider immunotherapeutic interventions.

Our proteome-based analysis identified HRD-associated proteins and an HRDness that could facilitate the adaptive and versatile identification of patients with YBC who may benefit from treatment with PARP inhibitors. Additionally, we demonstrated that protein–RNA correlations can be used to predict the late recurrence of hormone receptor-positive breast cancer. These findings have valuable clinical implications for improving survival outcomes and reducing treatment-related toxicity by guiding the optimal duration and selection of adjuvant treatment for patients with breast cancer. These findings can be clinically applied to guide the optimal duration and selection of adjuvant treatment for patients with breast cancer, which is particularly crucial for patients diagnosed with breast cancer at a young age. Within each standard molecular subtype of breast cancer, we identified functionally significant protein groups whose differential abundance closely correlated with the clinical progression of breast cancer. Moreover, we derived a recurrence-predictive cluster capable of predicting late recurrence, specifically for the luminal subtype, and validated its efficacy in various patient cohorts. Prognostication currently used for breast cancer depends largely on clinical subtypes and mRNA-based information. This proteome-integrated prognostic index plays a crucial role in guiding treatment duration decisions for early-onset breast cancer, thereby contributing to improved patient stratification and personalized treatment approaches.

## Supplementary information

Supplementary information

## Data Availability

All data that support the findings of this study are available from the corresponding authors upon IRB approval.
