# Supplementary material for: Proteogenomic analysis dissects early-onset breast cancer patients with prognostic relevance
Source: Exp Mol Med. 2024 Nov 1;56(11):2382–94. doi: 10.1038/s12276-024-01332-w (PMC11612404; doi:10.1038/s12276-024-01332-w)
Supplement: Supplementary file 1 — Supplementary information [file 12276_2024_1332_MOESM1_ESM.pdf]

Supplementary Information:

Supplementary Figures

Supplementary Fig. 1. A comparison of the dose-dependent effects of neratinib on HER-2, p-HER2 (Ser1054), and p-HER2 (Tyr1248) in HER2-positive breast cancer cell lines. SKBR3, MDA-MB-453, and BT-474 cells were treated with neratinib (10 and 50 nM) or DMSO for 4 h and 24 h. Immunoblot analysis was conducted for the indicated proteins. β-actin was used as a loading control. The representative results from three biological replicates are shown.

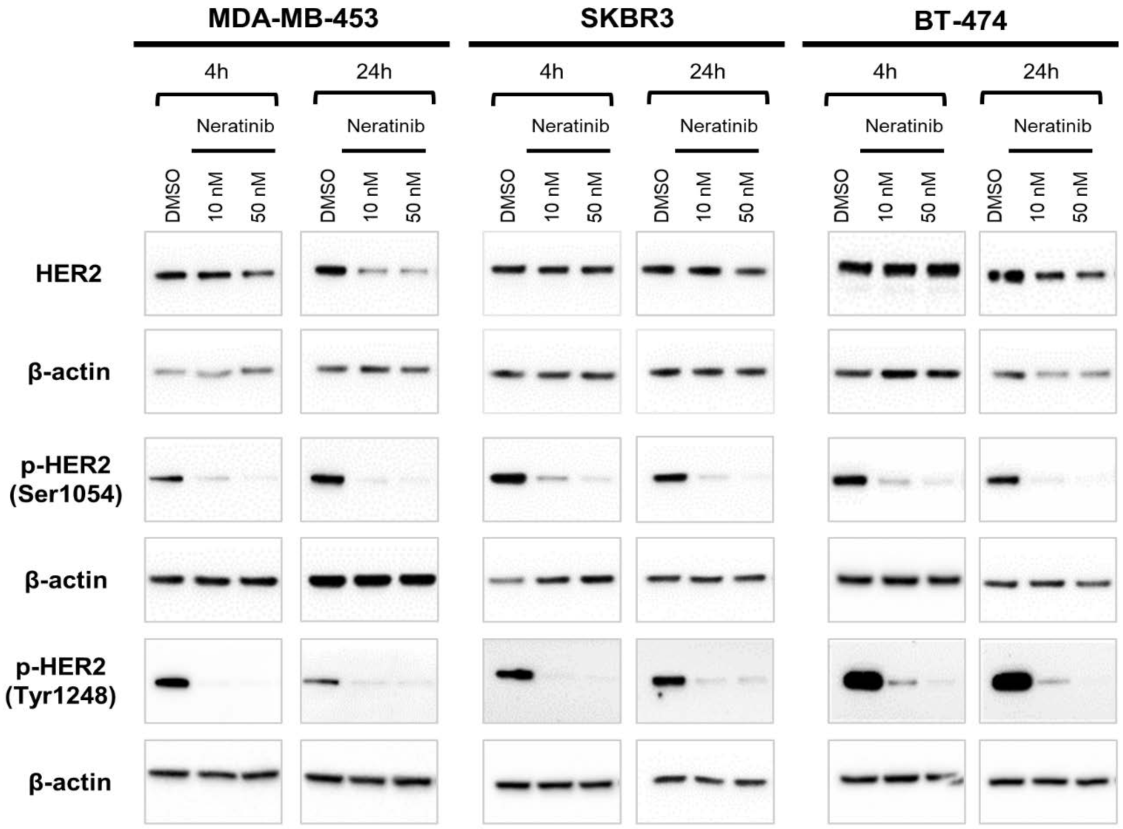

Supplementary Fig. 2. Correlation between proteogenomic (PG)-based immune signatures and the cell densities (cells/mm^2) for each immune marker or phenotype quantified by multiplex immunofluorescence staining. Spearman correlation coefficients are color-coded to indicate positive (red) or negative (blue) associations.

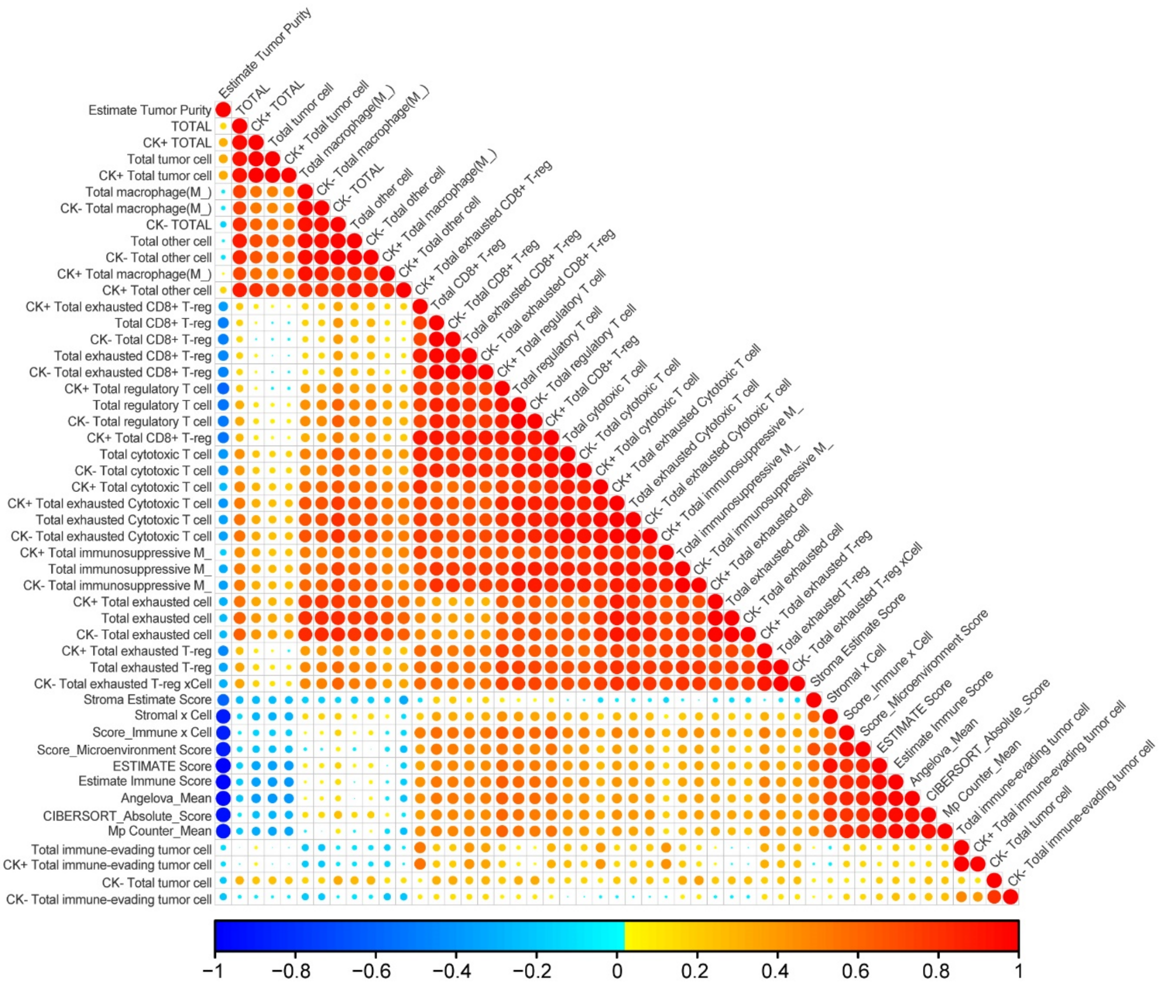

Supplementary Fig. 3. Heatmap showing the wide range of expression levels for immune-related features in each PAM50 subtype. The Z scores of RNA-based immune signatures from CIBERSORT, ESTIMATE, MCP counter, xCell, immune gene set from Angelova, as well as protein-derived signatures for immune modulator gene sets, are displayed in the top two data panels. The third data panel presents the scores for immune phenotypes, including T cells, CD8 T cells, Cytotoxic lymphocytes, B lineage, NK cells, Monocytic lineage, Myeloid dendritic cells, Neutrophils, Endothelial cells, and Fibroblasts.

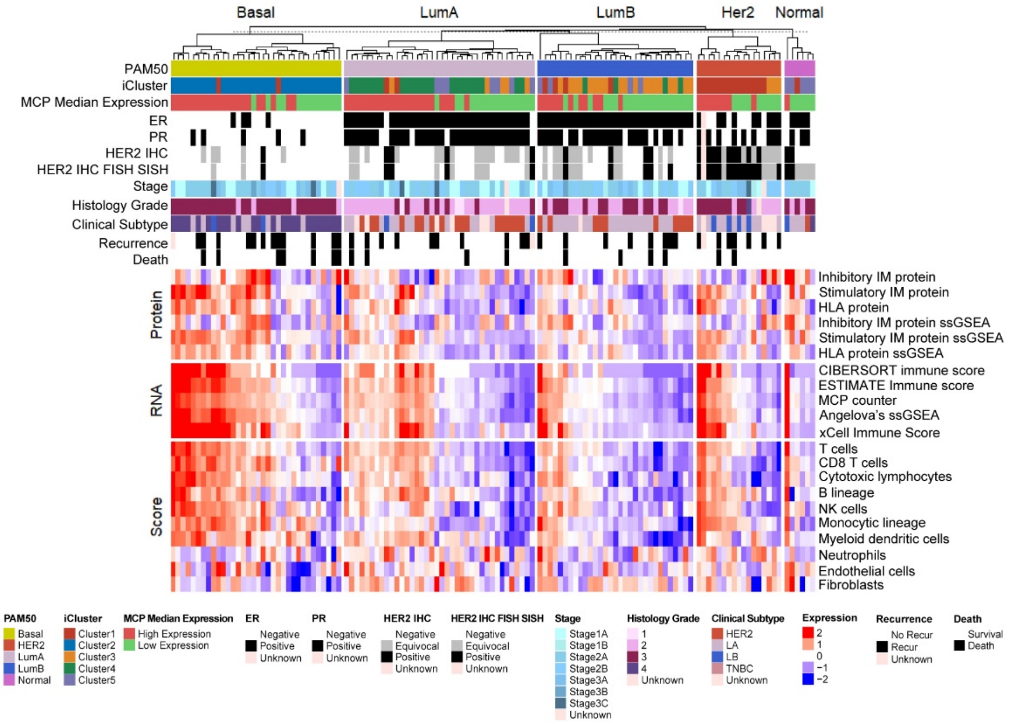

Supplementary Fig. 4. The estimated xCell immune scores in the PAM50 subtypes of the YBC cohort (ANOVA, *P* = 0.007).

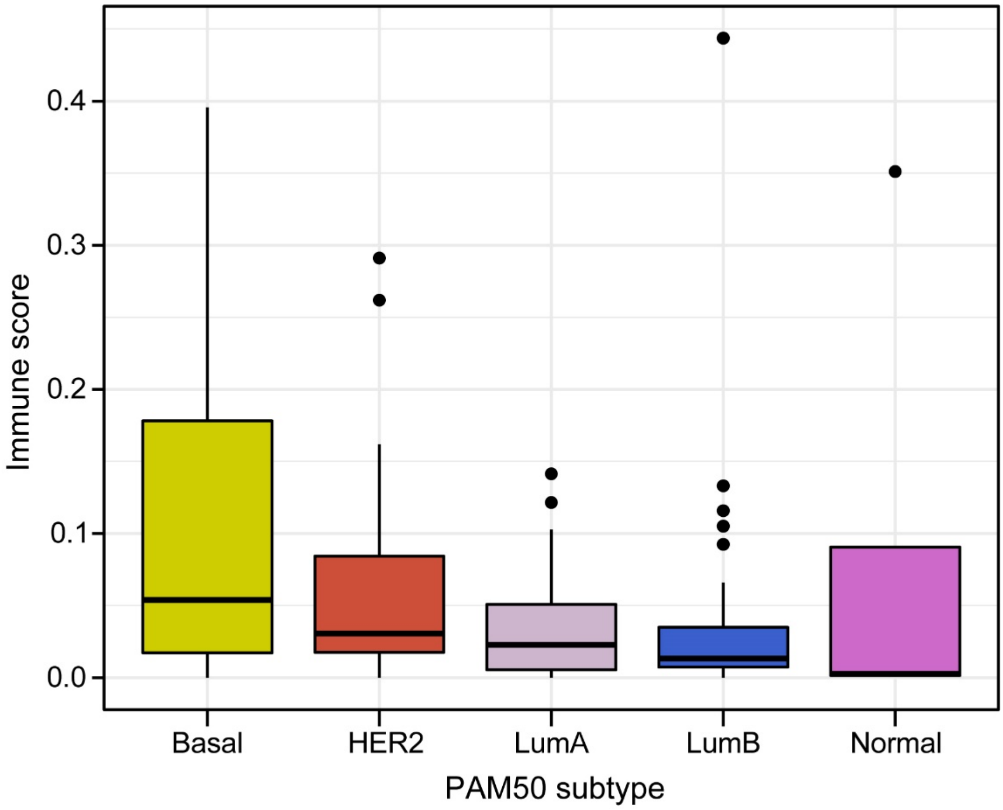

Supplementary Fig. 5. Distribution of absolute immune scores (CIBERSORT) in the YBC five integrative clusters (iClusters) (ANOVA, P = 0.001).

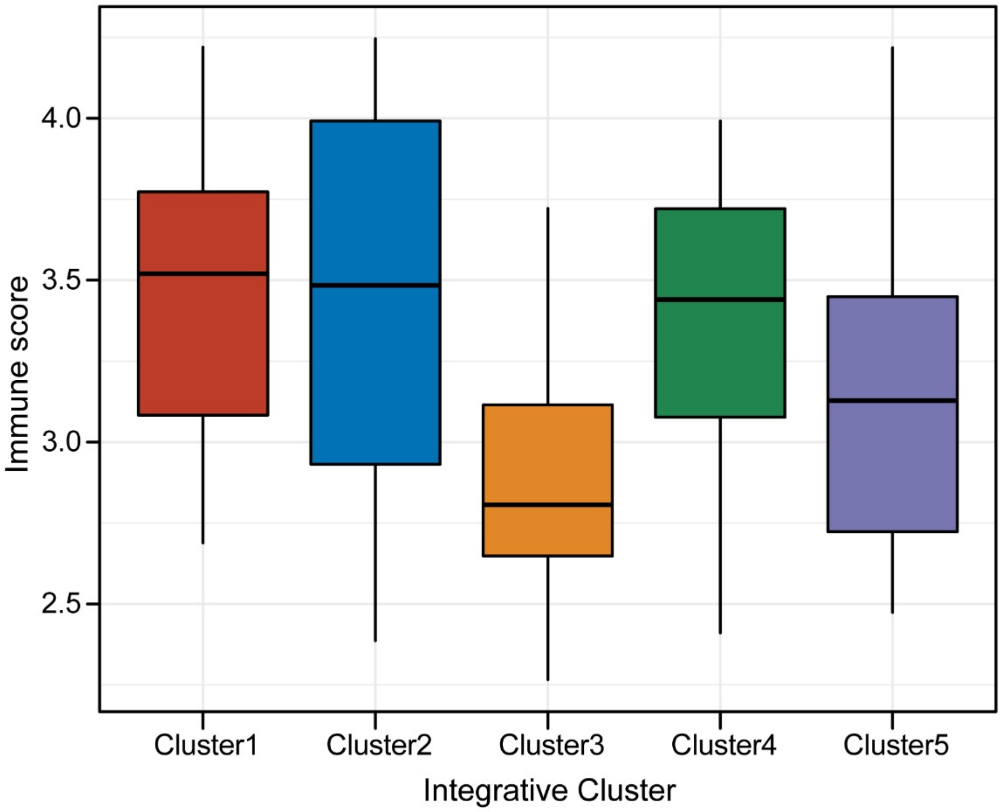

Supplementary Fig. 6. Kaplan-Meier (KM) plots showing progression free survival (PFS) based on the expression level (high vs. low) of the RNA-based immune signatures from CIBERSORT, ESTIMATE, MCP, and xCell algorithms in iCluster 3 patients. The threshold of “high” and “low” was based on the median expression level of immune signatures in iCluster 3 patients.

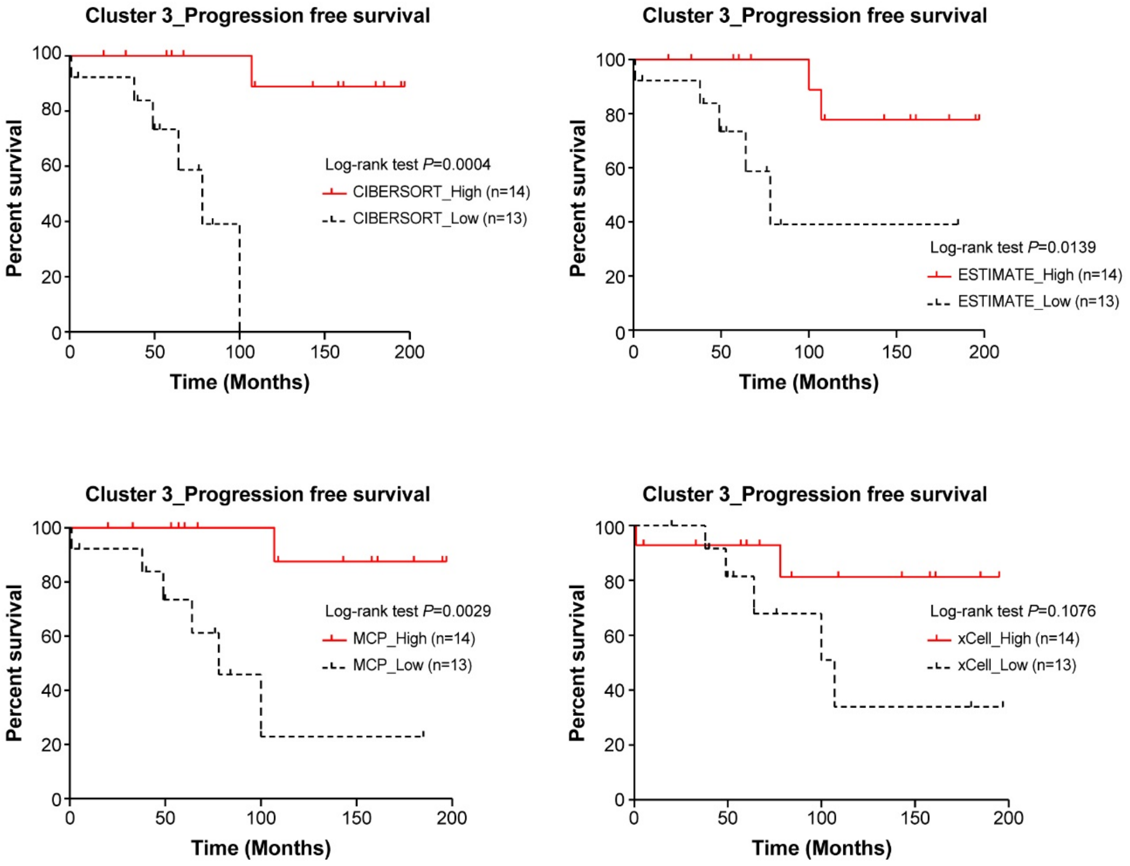

Supplementary Fig. 7. Protein-RNA correlation analysis. **a** Histogram depicting correlation between protein and RNA quantification values of individual genes. Red denotes a positive correlation between protein and RNA, while blue signifies a negative correlation. The lower panel represents the most highly correlated (top three lines) and the most uncorrelated (the bottom three lines) molecular pathways in YBC. ER: Estrogen receptor **b** KM survival curves demonstrate the predictive power of protein and RNA correlation in hormone-positive breast cancer. The subgroup with a high RNA-protein correlation shows a poorer prognosis compared to the subgroup with a low RNA-protein correlation (*p* = 0.0049).

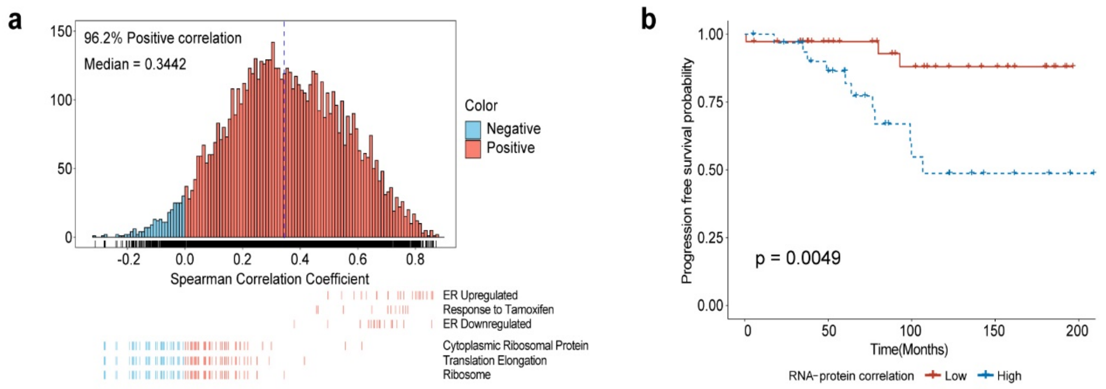

Supplementary Table

Supplementary Table 1. Clinical characteristics of patients with young breast cancer

**Characteristics**	**Total (N)**	**Total (%)**
No. of patients	126	100.00
Age at diagnosis (median, range)	37 (23-40)	
Age at diagnosis (Mean ± SD)	35.6±3.8	
T stage		
T1	40	31.7
T2	68	54.0
T3	15	11.9
T4	3	2.4
N stage		
N0	59	46.8
N1/N1mic	49	38.9
N2	10	7.9
N3	5	4.0
Nx	3	2.4
Stage		
I	27	21.4
II	76	60.3
III	20	15.9
IV	3	2.4
HG		
1	5	4.0
2	54	42.9
3	65	51.6
Unknown	2	1.6
ER		
Positive	84	66.7
Negative	41	32.5
Unknown	1	0.8
PR		
Positive	73	57.9
Negative	52	41.3
Unknown	1	0.8
HER2		
Positive	25	19.8
Negative	78	61.9

Unknown/ Equivocal	23	18.3
Ki-67		
<14%	43	34.1
≥14%	76	60.3
Unknown	7	5.6
Clinical subtype		
LA	33	26.2
LB	55	43.7
HER2	10	7.9
TNBC	25	19.8
Unknown	3	2.4
Surgery		
BCS	83	65.9
Mastectomy	40	31.7
Unknown	3	2.4
Chemotherapy		
Done	113	89.7
Not done	10	7.9
Unknown	3	2.4
Radiotherapy		
Done	104	82.5
Not done	19	15.1
Unknown	3	2.4
Target therapy		
Done	7	5.6
Not done	116	92.1
Unknown	3	2.4
Anti-hormonal therapy		
Done	89	70.6
Not done	33	26.2
Unknown	4	3.2
Follow-up periods (median, range (months))	94.3 (4.0-254.3)	
Recurrence		
Yes	37	29.4
No	86	68.3
Unknown	3	2.4
Death		
Yes	17	13.5
No	109	86.5

LA: luminal A, LB: luminal B, TNBC: triple-negative breast cancer, BCS: breast-conserving surgery
